# Effect of Coping Strategies Training On Its Use by Thalassemia Major Adolescents: A Randomized Controlled Clinical Trial

**Published:** 2015-01

**Authors:** Fatemeh Hashemi, Afie Naderi Darshori, Farkhondeh Sharif, Mehran Karimi, Najaf Zare

**Affiliations:** 1 Department of Pediatric Nursing, School of Nursing and Midwifery, Shiraz University of Medical Sciences, Shiraz, Iran;; 2 Community Based Psychiatric Care Research Center, Department of Mental Health and Psychiatric Nursing, School of Nursing and Midwifery, Shiraz University of Medical Sciences, Shiraz, Iran;; 3 Hematology Research Center, Shiraz University of Medical Sciences, Shiraz, Iran;; 4 Department of Biostatistics, School of Medicine, Infertility Research Center, Shiraz University of Medical Sciences, Shiraz, Iran

**Keywords:** Coping, Education, Thalassemia major

## Abstract

**Background: **Thalassemia is a chronic disease with serious clinical and psychological challenges. The incidence of thalassemia in a family member may cause a psychological crisis in all family members and in this case coping strategies are required. This clinical trial study aimed to determine the impact of training coping strategies on their use by major thalassemic adolescents referred to Dastgheib Hospital in Shiraz.

**Methods:**  In this randomized clinical trial, 87 adolescents with major thalassemia who were randomly assigned to two groups of experiment and control were enrolled. Then the experiment group attended six sessions of coping strategies training program in isolation, each lasting for one and half hour. In order to examine the coping strategies, Jalowice’s coping strategy questionnaire was used in three periods including pre-intervention period and one month and two months after the intervention. The collected data were analyzed using independent t-test and Chi-square.

**Results:** Mean scores of problem-focused coping strategies in the experiment group increased in one month and two months after the intervention from 45±12.7 to 54.8±7.3 and 55.7±7.2, respectively. Also, the difference in mean scores of problem-focused coping strategies was significant in the two groups (P<0.001).  Furthermore, the difference in mean scores of emotion-focused coping strategies was significantly different between the two groups in two months after the intervention (P=0.007).

**Conclusion: **Based on the obtained results, teaching coping strategies has improved the use of problem-focused coping strategies and also effective coping with stress and disease problems in patients with thalassemia major. Therefore, it is recommended that authorities should consider this as a part of treatment program.

**Trial Registration Number: **IRCT2013112215484N1

## Introduction

Thalassemia is an inherited blood disorder characterized by a defect in globin chain synthesis in red blood cells.^1^ It is a chronic disease that presents a range of serious clinical and psychological challenges.^2^ Long and undesirable treatment of thalassemia affects different aspects of patient’s life.^3^ Problems caused by this disease affect not only physical performance but also sensual, social and school functioning of thalassemia children.^2^ Thalassemia children feel that they are different from their peers and they have negative thoughts about life; they feel guilty, highly anxious, and low self-esteem^4^ Findings have shown that depression, anxiety and shyness are more common in thalassemic patients in comparison to healthy groups.^3^ On the other hand, adolescents suffering from chronic disease experience bilateral crisis; they should not only adjust to the complementary tasks but also, they need to  overcome  stress caused by disease including losing control, being different from peers, and death.^1^ Incidence of thalassemia in a family member may cause a psychological crisis in all family members and it requires coping strategies.^5^ Patient’s use of different strategies to overcome their problems and patients difference in using coping strategies show their difference in adjustment to stress.^6^ Coping is a process upon which people try to manage and handle the perceived discrepancy between the demands, requirements and resources they experience in stressful situations.^7^ Coping strategies are defined as mechanisms which people employ to master, tolerate and minimize or mitigate adverse effects of stressful events.^8^ They are generally divided into two types:^9^ first problem-focused coping strategies such as trying to establish control over the situation, gaining information about the problem, analyzing the problem into smaller components, determining specific goals to help solve the problem, using previous experiences to solve the problem, and talking to people with the same problem; and second emotion-focused coping strategies like hoping to improve the situation, praying, seeking help from family and friends, capitulating to fate, aggression, crying, medication intake, etc.^10^ Stress and its severity are not bad in itself but what is important is the way of dealing with this situation. Therefore, the strategy a person chooses is a part of the vulnerability profile.^11^ Effective active coping alleviates the problem and reduces emotional distress^12^ and using correct coping strategies can lead to positive results. Using inappropriate coping strategies in case of having tension increases the problems.^11^ The key to successful coping is using flexible coping strategies. Coping flexibility involves ability to change and adapt coping strategies over time and across different stressful conditions as different strategies work effectively than others depending on circumstances. Therefore, coping skills training increases the competence and mastery by retraining inappropriate or non-constructive coping styles and patterns of behavior toward the development of constructive behaviors.^13^ Therefore, teaching coping strategies to deal with the diseases is very useful.^10^ Patients with thalassemia should be able to employ those of coping strategies to enhance coping ability, so the ways to use these strategies should be taught. Since such a study hasn’t been conducted in Iran, this study was designed to determine the effect of teaching coping strategies on their use in major Thalassemic adolescents.

## Materials and Methods

**Figure 1:**
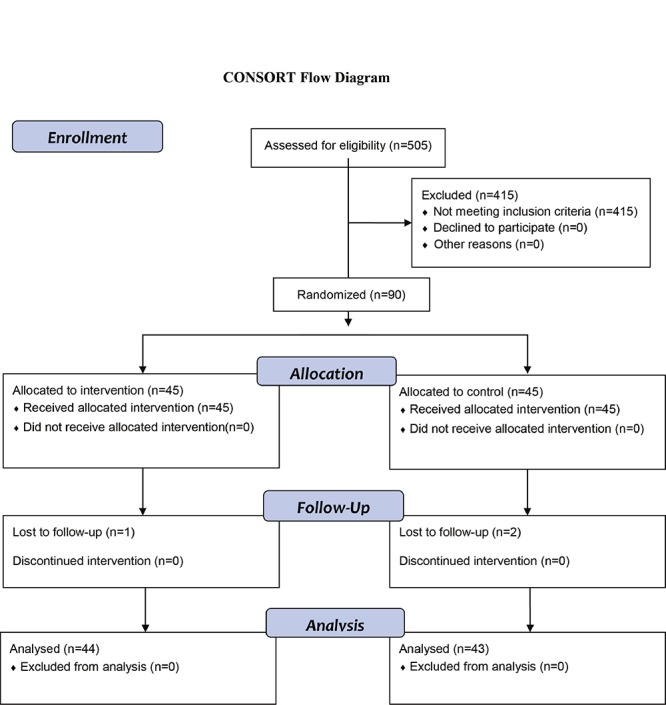
Diagram of the participants in the study

In the present randomized clinical trial, the effect of training coping strategies on using coping strategies in adolescent suffering from major thalassemia referred to Shahid Dastgheib hospital was considered. According to the findings of Aziznezhad et al.’s study and mean difference and reliability of 95%, power of 80%, SD of 10.5 and minimum acceptable difference of 6.5, 41 individuals were assigned into each group. Then by considering the attrition rate, 4 more individuals were added into each group. So each group consisted of 45 individuals. Next, patients with beta thalassemia major, who had referred to thalassemia center of Dastgheib in Shiraz from March 3^rd^ 2013 to September 9^th^ 2013, were examined in order to receive blood and 90 cases based on the inclusion criteria and by convenient sampling method entered the study. Inclusion criteria were age of 11 to 18 years old, being literate, having informed assent and consent for both teens and the parents in order to participate in the study, lack of chronic physical and mental illnesses according to information mentioned in patients’ records and physicians’ diagnosis, no acupuncture treatment, Yoga and/or complementary treatment, having a nuclear family (teen living with parents), and participating in all training sessions. Exclusion criteria were patient’s death, patient reluctance to continue working with the researcher during the study, diagnosis of another chronic physical or mental condition during the investigation by the patient’s physician or any stressful event for the patient and his/her family. Instruments for data gathering were demographic and Jalowice’s coping strategies questionnaires. Jalowice’s questionnaire contains 8 domains of coping with 39 questions- 24 statements about emotion-focused coping strategies and 15 about problem-focused coping strategies.^14^ All the scales assessed the patients’ strategies in a five point scale, ranging from 1-5 and from never to always. In Jalowice’s problem-focused coping strategies, the change  in scores in lieu of teaching from smaller to greater and also change in scores in emotion-focused coping strategies from greater to smaller in lieu of teaching the strategies was considered desirable.^10^ In Mahmoudi et al.’s study (2003), the validity of the questionnaire was determined by the expert faculty members and the reliability of the questionnaire was calculated and confirmed by retest method and alpha Cronbach 0.86.^15^ After explaining the objectives and research methodology to the patients and their parents, the patients’ assent and parents’ written consent to participate in this research were taken. In order to randomize the samples, simple randomization method was used.  For random assignment of the subjects to the experiment and control groups, random allocation table was used. In this way, cards with numbers 1 to 90 were provided and by using random number table, all the samples were assigned to two groups. Then, again by using a random number table, one group was selected as the control group and the other group as the experiment group. Then, demographic information form and Jalowice’s questionnaire were filled out through interviews with patients in both experiment and control groups. During the study, one patient from the experiment group and two from the control group were excluded from the study. Finally, the study was conducted on 87 participants (44 in the experiment group and 43 in the control group) (Figure 1). For the patient’s in the experiment group, training sessions about thalassemia, its treatment and complication, daily activities, physical activities, negative emotions and their control, reasonable thinking, stress and its side effects and managing stress and efficient coping strategies, during 6 sessions consisting of 1.5 hour every two weeks and for single patients (when referring to the hospital for blood transfusion) were held. The training content was presented in the form of lecture, question and answers by the researcher and a clinical psychologist holding MS degree. In case of need and based on the patients’ realization, training content was provided in a longer time. At the end of each session, they reminded the training session content to the experiment group patients to remember it better at home. In order to investigate studying training materials by patients between two sessions and reminding them, a review was done at the begging of each session. In order to avoid exchanging information between members of the two groups when they referred to the hospital, training sessions were arranged in different days and the experiment group patients were asked not to share research information with other people. Then, after one and two months of the sessions in order to determine the effectiveness of the intervention, the questionnaires were completed again by the cohorts (experiment and controls). Finally, the collected data were analyzed using the Statistical Package for the Social Sciences (SPSS16) according to the research objectives using independent t-tests, Chi-square.

## Results

The mean age of the patients in the experiment groups was 15±2.2 and it was15.4±2.5 in the controls (P=0.393). Fifty percent were female and 50% male in the experiment group and 48.8% were female and 51.2% were male in the control group (P=0.914). The experiment and control groups underwent oral chelating therapy with 47.7% and 58.1%, respectively (P=0.271). Considering the birth rank in the experiment and controls, 65.9 and 55.8 percent were firstborn or second sibling, respectively (P=0.181). Fifty percent in the experiment group and 60.5 percent in the control group were high school students (P=0.533). The two cohorts showed no statistically significant differences in terms of the above-mentioned points and the cohorts were similar (P>0.05). The Chi-square test didn’t prove a significant difference between educational level of the fathers (P=0.238) and mothers (P=0.520).  Regarding the father’s job (P=0.934) and mother’s job (P=0.598) and family income level (P=0.911), there were no significant differences between the experiment and control groups and they were similar from this point of view.

The problem-focused and emotion-focused coping strategies scores in the two groups before the intervention, using independent t-test, were not significantly different. The mean scores of problem-focused coping strategies in the cohorts within a month and two months after the intervention were different (P<0.001). Based on the results of independent t-test,  the mean scores of emotion-focused coping strategies one month after the intervention in the two cohorts were not statistically significant, but the difference between the scores of the cohorts two months after the intervention was approved (P=0.007) (Table 1).

**Table 1:** Comparison of mean scores of coping strategies in two studied groups in each phase of the study

**Independent t-test**	**Time**	**Before intervention **		**One month after intervention**		**Two months after intervention**		
**Variable**	**Group**	**Mean±SD**	**P value **	**Mean±SD**	**P value**	**Mean±SD**	**P value**
Problem-focused strategies	Experiment	45±12.7	0.664	54.8±7.3	<0.001	55.7**±**7.2	<0.001
	Control	46.1±10.5		46.2±10.3		45.7±10.3	
Emotion -focused strategies	Experiment	66.1±8.8	0.667	64.6±9.2	0.185	61.9±10.7	0.007
	Control	67±10.3		67.3±9.6		67.8±9.3	

P values lower than 0.05 are statistically significant

## Discussion

Mean scores of emotion-focused and problem-focused coping strategies in the two groups of experiment and control showed no statistically significant differences in the pre-intervention phase. Therefore, it can be presumed that coping strategies in the two groups before the intervention in terms of type and amount of the strategy used were identical. However, problem-focused coping scores in the cohorts of experiment and control were statistically significant in regard to the increase in problem-focused coping scores one month and two months after the intervention. The scores on problem-focused coping strategies enhanced, so based on the Jalowice’s questionnaire, changes from smaller to greater are desirable. The favorable change in the experiment group could indicate a positive impact of teaching coping strategies in this study. This means that patients were more willing to use problem-focused coping strategies after teaching. In line with the present study, Froozandeh et al. in a study aiming to investigate the effects of cognitive behavioral therapy on the coping strategies of non-medical students of Shahrekord University of Medical Sciences concluded that cognitive behavioral therapy in students has positive impacts on the use of efficient coping responses (problem-focused approach).^16^ The study of Lin Chen et al. on Chinese-American children demonstrated that the intervention group used more active coping strategies than the control group.^17^

Mean scores of emotion-focused coping strategies a month after the intervention, compared to before, decreased in the experiment group while those of the control group had a slight increase, but the difference was not statistically significant. However, two months after the intervention, the difference in scores in the cohorts in terms of emotion-focused coping strategies scores was statistically significant. So it can be concluded that teaching coping strategies could decrease the frequency of the use of emotion-focused coping strategies in the experiment group and consequently the scores of these strategies in comparison with the control group were lower after the intervention and this decrease in two months reached a statistical significance (P=0.007)(Table 1).

Madani et al. conducted a study on multiple sclerosis patients, indicating that self-care program is effective in reducing the use of emotion-focused coping strategies by patients.^18^ Gift and Austin in a study on patients with chronic obstructive pulmonary disease reported that after completion of the training programs, the experiment group used emotion-focused strategies less than the control group.^19^ The results of the study by Aziznezhad et al. entitled “Effect of self-care training on applying coping strategies of adolescents afflicted with thalassemia major” suggested that teaching increases the use of problem-focused coping strategies and decreases the use of emotion-focused coping strategies. But these values were not statistically significant. So, the authors believe that it takes time to change the coping strategies and also it requires more time so that the changes become habit.^10^ It can be said that the results of our study in a month after the intervention are in line with those of Aziznezhad et al.’s study. In spite of more reduction in the score of emotion-focused coping strategies in the experiment group in comparison with the control group, the statistical test showed no significant difference probably because of any significant difference in the scores between the cohorts. However, the results of our study differed from those of Aziznezhad et al.’s study two months after the intervention because the reduction in the score of emotion-focused coping strategies in the experiment group in comparison with the control group was statistically significant (P=0.007). There was a statistically significant difference in terms of emotion-focused coping strategies score in both groups, after two months of intervention in comparison with one month after the intervention, which showed no significant difference. This could be due to one extra month of the experiment group so that more behavioral changes were observed in this group after two months. However, the difference between the present study and that of Aziznezhad et al, regarding the significance of the scores in the experiment and control groups two months post-intervention, could be related to differences in the educational content of the studies. In the study of Aziznezhad et al, self-care skills were taught,^10^ while in the current study coping strategies were provided and taught for the experiment group; also, the difference between the two studies could be due to the number and hours of training sessions or teaching methods. In our study, the experiment group underwent a total of 9 hours of lecture, and question and answer in six sessions. While in Aziznezhad et al.’s study, the experiment group attended five sessions of 45 minutes.^10^

The results of the study of Inouye et al. showed that using self-management training in individuals with HIV/AIDS can reduce some of emotion-focused coping strategies.^20^ Also the results of the study of Gil et al. on subjects with sickle cell anemia showed that training cognitive coping skills may increase coping attempts and decrease negative thinking.^21^

So it could be concluded that teaching coping strategies to the experiment group could increase the behaviors associated with problem-focused coping strategies and decrease the behaviors associated with emotion-focused coping strategies. This is a positive and desirable result of teaching the Jalowice’s questionnaire because increase in the use of problem-focused coping strategies can lead to effective and positive confrontation of thalasemic adolescents with the complications of the diseases and as a result can have a positive impact on the quality of life.

Generally, it can be concluded that teaching coping strategies can increase the use of problem-focused coping strategies along with a decrease in emotion-focused coping strategies use in adolescents with thalassemia major. 

## Conclusion

The findings of this study revealed that teaching coping strategies improved effective coping skills to confront stress and related complications with the disease in patients with thalassemia major. Therefore, it is recommended that this kind of teaching should be considered as part of treatment programs. So, establishing infrastructure of counseling clinic in hospitals which admit patients with thalassemia, determining the roles and responsibilities of consultants and defining nurses’ status as counselors at the clinics are required. 
